# Suitability of Nasal and Deep Nasopharyngeal Swab Sampling of Calves in the *Mycoplasma bovis* Control Program

**DOI:** 10.3389/fvets.2021.689212

**Published:** 2021-09-10

**Authors:** Tarja Pohjanvirta, Nella Vähänikkilä, Vera Talvitie, Sinikka Pelkonen, Tiina Autio

**Affiliations:** ^1^Veterinary Bacteriology and Pathology, Finnish Food Authority, Kuopio, Finland; ^2^Department of Production Animal Medicine, Faculty of Veterinary Medicine, University of Helsinki, Saarentaus, Finland; ^3^Animal Health Ettry, Seinäjoki, Finland

**Keywords:** *Mycoplasma bovis*, diagnostics, nasal swab, deep nasopharyngeal swab, control program, calves, cattle

## Abstract

*Mycoplasma bovis* is an important cattle pathogen affecting animal health, welfare, and productivity. The main disease syndromes are mastitis, pneumonia, and otitis media in young stock, as well as arthritis. Response to antibiotic treatment is poor and no effective vaccine is available. Asymptomatic carriers are common and usually harbor the organism in the airways or mammary glands. Purchase of carrier animals is a major risk for the introduction of infection into naive herds. Following the detection of *M. bovis* in Finland in 2012, a voluntary control program was established. It aims to prevent the spread of the infection and to help farms attain certification of a low *M. bovis* risk. Among the diagnostic tools in the program, nasal swabs (NS) from young calves have been tested for *M. bovis* to indicate the infection status of the herd. In this study, we assessed the suitability of this test method. We analyzed the effectiveness of NS and deep nasopharyngeal swabs (NP) to detect *M. bovis* in pneumonic and healthy calves in dairy herds recently infected with *M. bovis*. In pneumonic calves, NP sampling followed by culture and real-time PCR demonstrated a proportion of positive agreement (PPA) of 0.91 compared with bronchoalveolar lavage (BAL), whereas NS showed only 0.5 PPA compared with BAL. Among healthy dairy calves, overall *M. bovis* prevalence in NS was 29.6%. The highest rate of shedding (43%) occurred in calves 31–60 days old. At the calf level, *M. bovis* prevalence in NP samples was 47% compared with 33% in NS samples among the 284 studied calves. However, at the herd level, NS sampling classified 51 out of 54 herds with a positive infection status as infected, whereas in NP sampling, the respective figure was 43 out of 54 herds (*p* = 0.061). In conclusion, NS sampling from calves under 6 months of age and analyzed by real-time PCR is a cost-efficient method for a control program to detect *M. bovis* in dairy herds, even if no *M. bovis* mastitis has been detected in the herd. For pneumonic calves, we recommend only NP or BAL sampling.

## Introduction

*Mycoplasma bovis* is increasingly recognized as a significant disease-causing agent in various age groups of cattle. The infection presents itself with different signs, such as pneumonia, which mainly occurs in young animals, mastitis, arthritis, otitis media, and rarely keratoconjunctivitis or reproductive tract problems (1, 2). *M. bovis* infections tend to be chronic and the response to antibiotic therapy is often poor (2). There is increasing resistance to the antibiotics commonly used to treat pneumonia in calves in Europe (3–5), and no effective commercial vaccine is available (6). *M. bovis* infections have a debilitating effect on animal welfare and can be costly to farmers. There is a critical need to develop preventive measures to reduce the effects of *M. bovis* infections in the cattle industry. One such a preventive measure could be a control program aiming to reduce the risk of introducing *M. bovis* into naive herds through animal trade. *M. bovis* was detected in Finland for the first time at the end of 2012 (7). During 2013, a voluntary control program was established by Animal Health ETT and the cattle industry. The aim of the program is to ensure that dairy and suckler cow herds in the highest level of the program are free of *M. bovis*, and thus prevent the spread of the agent between herds when live animals are purchased. This also relieves the *M. bovis* infection pressure and reduces the use of antimicrobials in specialized calf-rearing farms, as their calves originate from dairy farms.

Several methods need to continuously be applied to ensure that a dairy herd is free of *M. bovis*. The main elements in the Finnish *M. bovis* control program are regular herd health visits, clinical monitoring, and sampling of suspected cases, such as calf pneumonia, routine testing of mastitis samples, nasal swab sampling of healthy calves, and the control of animal trade and movement. The main manifestation of *M. bovis* in cows is mastitis. In Finland, individual clinical and subclinical mastitis milk samples are extensively tested. Almost all milk samples are tested using a multiplex PCR assay. In this test, specific primers to detect *M. bovis* have been in use since the beginning of 2012. However, in some dairy herds, *M. bovis* can cause pneumonia in calves without causing mastitis in cows, or only in few cows, which may remain undetected (7). Consequently, even rigorous mastitis milk sample testing to detect *M. bovis* does not guarantee that a herd is free of the agent. Testing for *M. bovis* antibodies was not regarded as a useful tool in the control program because of the low sensitivity and specificity of the ELISA tests previously available (8). Thus, we could not confidently rely on serological testing of herds to detect subclinical infections.

Different anatomical sites for the detection of *M. bovis* in carrier animals have been studied, but no site has been found that could be consistently used. In an earlier study by Bennet and Jasper (9), *M. bovis* was significantly more often found in the nasal secretions of healthy young calves in herds with *M. bovis* mastitis compared with non-infected herds. Based on this finding, nasal swabs (NS) taken from calves up to 6 months of age and analyzed by real-time PCR for *M. bovis* were included in the program. NS are affordable and practical to use in the field, as swabbing of young calves is relatively easy and quick. Another more cumbersome and more expensive technique would be deep nasopharyngeal swabs (NP) to sample pharyngeal lymphoid tissue. It has previously been shown that in young calves, *M. bovis* can colonize the tonsils without nasal shedding (10). However, to our knowledge, the suitability of NP to detect *M. bovis* in pharyngeal lymphoid tissue in healthy dairy calves has not been investigated.

The objective of this study was to (1) determine the overall apparent prevalence of nasal shedding in calves in dairy herds with recently confirmed *M. bovis* infection, (2) study the apparent *M*. *bovis* prevalence in nasal swabs (NS) in different age groups of calves under one year of age, (3) assess the suitability of NS and NP sampling at the herd level to detect carrier calves, and (4) compare different sampling and analytical methods to detect *M. bovis* in calves with acute respiratory disease.

## Materials and Methods

### Calves With Acute Respiratory Disease

Two veterinarians clinically evaluated 62 non-vaccinated calves aged 3–22 weeks with acute respiratory disease signs in two calf-rearing farms with endemic *M. bovis*. The veterinarians took NS, NP, and bronchoalveolar lavage (BAL) samples from each calf. These calves had not been medicated with antibiotics during the month before sampling.

### Calves in Dairy Farms Recently Infected With *M. bovis*

Clinically healthy calves aged from 3 to 348 days in 30 *M. bovis*–infected dairy herds were included in the study. Herds 1 to 19 and their *M. bovis* infection status were described in Vähänikkilä et al. (7). These herds were sampled by a veterinarian four times at approximately 6-month intervals. Two more dairy herds (herds 20 and 21) were sampled twice with a 6-month interval, and nine herds (herds 22–30) were sampled once. During each visit, the veterinarian took NS from a maximum of 20 (range 6–23, depending on the herd size) of the youngest calves on the farm. In addition, NP samples were also collected from five calves per herd and per visit. The number of cows in the study herds varied from 18 to 315, the mean being 91 cows, and 9/30 herds had 100 or more cows (Table 1). We included in this analysis the results from the first visit to each farm, and thereafter the results from the visits where at least one positive NS or NP was found in the herd, meaning that the infection status of the herd was then positive.

**Table 1:** Agreement between bronchoalveolar lavage (BAL) and nasal swab (NS) or deep nasopharyngeal swab (NP) in detecting *M. bovis* in 62 calves with acute respiratory disease.

**Sample**	**Detection method**	**Number of calves with each combination**				**Proportion of positive**	**Kappa (95% CI)**	***P* (kappa)**
		**of results (BAL/compared method)**				**agreement**		
		**+/+**	**+/–**	**–/+**	**–/–**			
NS	Culture	15	14	0	33	0.68	0.53 (0.34, 0.72)	0.000
NS	Real-time PCR	14	15	0	33	0.65	0.50 (0.31, 0.69)	0.000
NP	Culture	24	5	0	33	0.91	0.84 (0.7, 0.97)	0.000

As soon as possible after *M. bovis* diagnosis, farmers were given advice to apply measures aiming to prevent the spread of infection, described in detail in Haapala et al. (11). Briefly, the farmers were advised to separate newborn calves from the dam immediately after birth into a clean pen in a space separate from older animals. Unpasteurized colostrum was fed to all calves, followed by milk replacer or raw milk from healthy cows. None of the farms bought colostrum from another farm.

### Sampling Techniques

Nasal swabs (Transystems, Copan, Brescia, Italy) were taken prior to NP and BAL. The nostrils were cleaned with a paper towel and the swab was inserted into a nostril to a depth of ~13 cm. Two nasal swabs, one for PCR and one for mycoplasma culture, were simultaneously collected from calves with acute respiratory disease and one NS was taken from healthy calves in dairy herds. NP swabs were taken with 27-cm-long guarded swabs (Medical Wire Equipment Ltd, Corsham, England). The sheathed swab was inserted into the ventral nasal cavity approximately 1 cm rostral to the medial canthus of the eye, and the swab was advanced a few centimeters to the nasopharynx area and rotated. The swab was withdrawn into the sheath before removal. BAL samples were collected using a self-made double-guarded plastic catheter inserted through the nose into the trachea. Then, the inner catheter was pushed out and advanced until it wedged in a bronchus. Thirty to forty milliliters of sterile 0.9% saline was injected and immediately aspirated back into the syringe (12). The swabs intended for mycoplasma culture were soaked in D broth (13) and 0.5 ml of the BAL sample was transferred into D broth. The samples were transported to the laboratory within 24 h in styrofoam boxes with a freezer pack.

### M. bovis Culture

A 10-fold dilution from D broth to F broth (14) was made, and tightly closed tubes were incubated at 37 °C for 3–5 days to enrich *M. bovis*, followed by identification of *M. bovis* using real-time PCR (7).

### *M. bovis* Real-Time PCR

DNA was extracted from nasal swabs according to Sachse et al. (15). Real-time PCR was performed as described (7). The cut-off value for *M. bovis*–positive real-time PCR results was set to C_t_ 37.0.

### Statistical Analysis

We evaluated the agreement among sampling and detection methods by calculating the proportion of positive agreement (PPA), the kappa coefficient, and the corresponding *p*-value for kappa using Epitools Epidemiological Calculators (16). The kappa coefficient was interpreted according to McHugh (17): 0–0.20 no agreement, 0.21–0.39 minimal, 0.40–0.59 weak, 0.60–0.79 moderate, 0.80–0.90 strong, and above 0.90 almost perfect agreement. To determine whether NS and NP sampling differed significantly in the ability to assess a herd visit as positive, McNemar's χ^2^ test was conducted (16). Significance was set at *p* < 0.05.

## Results

### Detection of *M. bovis* in Calves With Acute Respiratory Disease

*M. bovis* was detected in 29/62 (47%), 24/62 (38.7%), 15/62 (24.2%), and 14/62 (22.6%) of BAL, NP, NS (culture), and NS (real-time PCR) samples, respectively. The proportion of positive agreement of NP compared with BAL was 0.91 and the kappa coefficient was 0.84 (strong), whereas the proportion of positive agreement of NS (real-time PCR) compared with BAL (culture) was 0.65 and the kappa coefficient was 0.50 (weak) (Table 1). Nasal swabs analyzed by culture only yielded one more positive sample compared with PCR from swabs (Table 1). NS (real-time PCR) proportion of positive agreement compared with NP culture was 0.68 and kappa coefficient was 0.53 (weak) (Table 2).

**Table 2:** Agreement between deep nasopharyngeal swab (NP) culture and nasal swab (NS) PCR in detecting *M. bovis* in dairy herd calves (*n* = 284) and calves with respiratory disease (BRD, *n* = 62).

**Sample**	**Detection method**	**Number of calves with each combination**				**Proportion of positive**	**Kappa (95% CI)**	***P* (kappa)**
		**of results (NP culture/NS real-time PCR)**				**agreement**		
		**+/+**	**+/–**	**–/+**	**–/–**			
NS (dairy)	Real-time PCR	77	56	16	135	0.68	0.48 (0.38, 0.58)	0.000
NS (BRD)	Real-time PCR	13	11	1	37	0.68	0.56 (0.35, 0.77)	0.000

### Calves in Dairy Herds Recently Infected With *M. bovis*

The total number of NS taken from 3- to 348-day-old calves was 1,037. The overall apparent *M. bovis* prevalence in nasal swabs was 29.5%. The highest prevalence of 43% was detected in calves aged 31 to 60 days. Thereafter, shedding decreased and was 13.7% in 150- to 180-day-old calves (Table 3). Large variation from zero to 75% was seen between the herds in the apparent prevalence of nasal shedding. Both NS and NP samples were taken from 284 calves. *M. bovis* was detected in 93/284 (32.7%) and in 133/284 (46.8%) of NS and NP samples, respectively. Proportion of positive agreement of NS compared with NP samples in these calves was 0.68 and the kappa coefficient was 0.48 (weak) (Table 2).

**Table 3:** Number of nasal swabs (NS) sampled from 30 dairy herds and number (%) of *M. bovis* PCR-positive swabs per age group (*d* = age in days).

**Age (*d*)**	**Number sampled**	**Number (%) of PCR-positive NS**
0–30	259	67 (25.9)
31–60	260	112 (43.1)
61–90	199	62 (31.2)
91–120	103	23 (22.3)
121–150	105	22 (21.0)
151–180	51	7 (13.7)
181–348	60	13 (21.7)
Total	1,037	306 (29.5)

### Effectiveness of NS and NP Samples in Indicating the Infection Status of Dairy Herds

Altogether, there were 54 herd visits with a positive infection status in which at least one positive NS or NP was found in the herd (Tables 4, 5). All samples from two herds were already negative at the first visit, and during one visit, only NS samples were taken from herd D (Table 4). Out of the 54 herd visits with a positive infection status, 51/54 (94.4%) would have been classified as infection status positive if only NS had been analyzed, and 43/54 (79.6%) as infection status positive if only NP samples had been analyzed (Tables 4, 5). This difference was not statistically significant (*p* = 0.061).

**Table 4:** *M. bovis* detected in nasal (NS) and deep nasopharyngeal (NP) swabs in 30 dairy farms during the first visit after the index case.

**Herd ID**	**Number of cows**	Index case^*^	**Days from index case to first sampling**	**No. positive/total**	
				**NS**	**DNP**
1	47	M	185	2/19	2/5
2	61	M	23	10/19	4/5
3	183	M	12	10/20	4/5
4	268	M	56	17/50	ND
5	25	M	79	9/15	3/5
6	50	M	16	4/11	1/5
7	157	M	36	10/20	3/5
8	60	M	37	3/15	3/5
9	100	M	41	2/20	1/5
10	61	M	30	14/20	4/5
11	29	M	112	0/7	0/5
12	41	M	12	7/20	6/6
13	66	M	74	0/10	0/5
14	158	M	27	9/23	2/5
15	48	CP	27	4/16	0/6
16	18	M	23	1/6	0/5
17	28	M	22	2/19	0/5
18	66	CP	48	5/20	3/5
19	127	M	21	8/11	5/5
20	94	M	93	1/14	5/5
21	84	M	8	8/20	4/5
22	315	NS	8	10/20	4/5
23	48	M	142	7/11	5/5
24	223	M	39	12/16	1/5
25	30	M	2	0/6	3/5
26	140	M	60	7/19	3/5
27	78	CP	14	4/20	1/4
28	60	CP	7	6/28	3/4
29	44	M	13	3/14	5/5
30	25	NS	47	5/20	4/5
Total				180/529 (34%)	79/145 (54.5%)

* *M, mastitis; CP, calf pneumonia; NS, nasal swabs taken to join the M. bovis control program; ND, not done*.

**Table 5:** *M. bovis* in nasal (NS) and deep nasopharyngeal (DNP) swabs during visits 2–4 to farms with a positive infection status.

	**Visit 2**		**Visit 3**		**Visit 4**	
	**No. positive/total**		**No. positive/total**		**No. positive/total**	
**Herd ID**	**NS**	**DNP**	**NS**	**DNP**	**NS**	**DNP**
1	1/15	2/6	1/21	0/5	–	–
2	3/24	0/5	–	–	–	–
3	12/20	3/5	3/19	4/5	1/20	0/5
4	10/20	2/5	2/20	2/10	7/22	1/5
5	0/10	1/5	4/13	5/5	–	–
6	2/18	2/5	–	–	–	–
7	–	–	3/19	0/5	–	–
8	9/19	3/5	–	–	1/19	0/5
9	6/18	4/5	1/20	0/5	–	–
12	–	–	9/20	3/5	7/16	0/5
14	14/25	4/5	8/20	4/5	–	–
18	–	–	–	–	10/21	5/5
19	6/20	4/5	3/20	3/5	0/15	1/4
20	2/20	1/5	^*^	^*^	^*^	^*^
22	1/14	0/5	^*^	^*^	^*^	^*^
Total	66/223	26/61	34/172	21/50	26/113	7/29

*– Infection status of the farm negative, all NS and NP negative*.

* *No visit*.

## Discussion

We assessed the suitability of nasal swab (NS) and deep nasopharyngeal swab (NP) sampling of young calves for use in the *M. bovis* control program. We observed an apparent overall *M. bovis* prevalence of 29.5% in NS sampling, with the highest prevalence of 43% in 31- to 60-day-old calves. At an individual level, NP sampling was the most sensitive sampling method for detecting *M. bovis* in healthy calves under 6 months of age. However, at the herd level, NS sampling was slightly more efficient than NP sampling in healthy young stock. Real-time PCR from NS correctly classified 51/54 herd visits with a positive infection status as positive, in contrast to NP sampling, which classified only 43/54 visits correctly. However, the difference only approached statistical significance (*p* = 0.061). The reason for this difference is related to the sampling protocol, as we took NS from the 20 youngest calves in the herd during the visits and only five NP samples. Guarded NP swabs are expensive compared with simple bacteriological swabs used in nasal swabbing, and an assistant is needed to restrain the calf's head when a NP sample is taken. By taking several NS samples from young calves, the sensitivity of the sampling method increases, which allows its use as a cost-efficient method in the *M. bovis* control program.

There have only been a few reports on the prevalence of nasal shedding of *M. bovis* in dairy calves. Bennet and Jasper (8) observed that approximately 34% of calves in herds with *M. bovis* mastitis shed *M. bovis* in nasal secretions compared with 6% of calves in non-mastitis herds. In our study, the overall prevalence of nasal shedding in dairy herd calves during the first visit after a confirmed *M. bovis* index case was the same as in the study of Bennet and Jasper (8), namely, 34%. Interestingly Bennet and Jasper (8) found that in *M. bovis*–infected herds, the highest prevalence of nasal shedding occurred in calves at around 5 weeks of age, and in general in various age groups, nasal shedding was highest at between 1 and 4 months of age. In our study, the highest prevalence of 43% was detected in calves aged 4 to 8 weeks, and thereafter the prevalence declined, being only slight under 14% in 22- to 26-week-old calves. However, in our study, only a small fraction of nasal swabs was taken from calves older than 5 months. Therefore, some caution is necessary regarding the prevalence in older calves. Other studies have reported a substantially lower nasal prevalence. In Denmark, Feenstra et al. (18) determined that 18% of nasal swabs from 0 to 6-month-old calves in herds with *M. bovis* mastitis were positive compared to 11% from non-mastitis herds. Recently, in Australia, Hazelton et al. (19) followed 450 heifer calves in eight herds, seven of which were *M. bovis* mastitis herds, and found that at weaning, only 2.4% of the calves were shedding *M. bovis* into nasal secretions.

Several factors might affect the observed nasal shedding prevalence of *M. bovis*. One is the detection method. All previous studies (9, 18, 19) have used a plate culture method, whereas we used real-time PCR. Our *oppD* real-time PCR and culture method both displayed an analytical sensitivity of 10^2^ cfu/ml *M. bovis* in BAL fluid (20). In this study, we also compared real-time PCR and culture results from nasal swabs taken simultaneously from pneumonic calves. The culture method detected only one more positive sample compared with real-time PCR. However, PCR analysis is available in many laboratories, whereas mycoplasma culture is demanding and only available in specialized laboratories, thus making PCR a more useful method in a control program. Another factor that affects the *M. bovis* prevalence in calves is the housing conditions. If newborn calves are isolated from older, presumably infected animals in another building or outside hutches, and cows with *M. bovis* mastitis are culled, it is likely that the calves will display no nasal shedding of *M. bovis* (11).

Previously, Maunsell et al. (10) have demonstrated that after oral inoculation of *M. bovis*, both palatine and pharyngeal tonsils were the main site of *M. bovis* colonization. However, despite heavy colonization of the tonsils, only two out of eight calves in the experiment were found to shed *M. bovis* into nasal secretions. Thus, the tonsils, rather than the epithelium of the nasal passages, may be the main upper respiratory tract colonization site, and tonsil swabs may be the best sampling method to detect *M. bovis* colonization. Data supporting this were also reported following a study by Haapala et al. (21), in which the tonsils of 4 out 20 clinically healthy bovines were colonized. NP swabbing samples the respiratory and associated lymphoid epithelium of the nasopharynx. We therefore compared *M. bovis* detection from NS and NP taken from 284 calves under 6 months of age in our study herds. At the individual level, NP sampling detected more positive calves (133/284) than NS (93/284). This suggests that tonsillar (in this case pharyngeal tonsillar area) swabs are indeed more sensitive than nasal swabs in detecting *M. bovis* colonization. This finding was recently confirmed by Buckle et al. (22) in New Zealand. They analyzed palatine tonsillar swabs taken at slaughter from healthy 3- to 5-month-old calves from a *M. bovis* seropositive herd. Real-time PCR detected *M. bovis* in almost 93% of the tonsillar swabs, whereas only 12% of tracheal swabs were positive. In the studies of both Maunsell et al. (10) and Buckle et al. (22), tonsillar swabs were taken *post mortem*. Swabs from the tonsil crypts are difficult to take from live animals, and the most comparable technique is NP sampling.

A high tonsil colonization rate of calves can be expected in herds in which *M. bovis* mastitis milk or contaminated colostrum is fed to calves (10). Studies on the prevalence of *M. bovis* in colostrum are scarce and the topic has not been investigated in Finland. Gille et al. (23) examined colostrum samples from 17 herds recently infected with *M. bovis* using PCR detection. In only four herds out of 17, *M. bovis* DNA was detected in 1.9% of colostrum samples. In some samples, borderline C_t_ values were recorded, and it is unclear whether these colostrum samples contained enough bacteria to infect the calf. Timonen et al. (24) estimated the *M. bovis* prevalence in colostrum to be 1.7–4.7% in four very large Estonian dairy herds in which *M. bovis* mastitis cows were not always culled. In Finland, colostrum is given to calves unpasteurized. It is possible that some of the calves in our study herds became colonized through colostrum. Approximately 170,000 individual clinical and subclinical mastitis quarter milk samples are tested annually in Finland (there are approximately 260,000 dairy cows in the country). Cows with *M. bovis* mastitis are segregated from the milk herd and are usually rapidly culled or slaughtered (7). The feeding of mastitis milk to calves in our study herds was highly unlikely, as the farmers were strictly advised not to give any mastitis milk to calves. Thus, pharyngeal colonization observed in our study is more likely to be characteristic of *M. bovis* infection in young calves rather than a result of feeding contaminated milk to calves.

The classification of a herd as *M. bovis* positive or negative is difficult and requires different sampling strategies and tools for different animal groups. The average herd size of dairy herds in Finland is 50 cows (25). As nicely demonstrated in the study by Humphry et al. (26), an imperfect test applied to a small herd is problematic. Testing of clinical mastitis and respiratory disease cases in calves is an essential part of the control program. Testing of *M. bovis* antibodies has demonstrated that the infection spreads rapidly in the herd and high antibody levels persist in cows for a long time (7, 27). Thus, testing of antibodies is not suitable to detect active infection. Moreover, previous studies have demonstrated that during an initial outbreak of *M. bovis* mastitis, colonization and shedding are not consistently associated with a particular anatomical site and shedding rapidly decreases in cows (28, 29). Thus, NS sampling of cows is not efficient for a control program. However, in calves, *M. bovis* is more prevalent in the upper respiratory tract, and NS sampling should be targeted at calves. The number of swabs taken from a control program herd during each sampling should be based on the average number of calves available for sampling and should aim to keep laboratory analysis costs reasonable. In our program, the herd health veterinarian visits each herd biannually and sampling is included in these visits. Biannual sampling allows targeting at calves younger than 6 months old in which *M. bovis* prevalence is at its highest.

We compared different sampling and detection methods in pneumonic calves to determine the most cost-effective method to sample clinical cases in herds in the control program. In pneumonic calves, NP had a strong agreement with BAL sampling in detecting *M. bovis*. Recently, Doyle et al. (30) examined the agreement among four sampling methods in the detection of different bovine respiratory disease pathogens. They compared the agreement of NS and NP with transtracheal wash in pneumonic dairy calves aged 31–74 days. Plate culture and PCR identification was used to detect *M. bovis*. Their study yielded a very good positive agreement of 91 and 92% and a kappa value of 0.82 and 0.83, respectively, when NS and NP were compared with transtracheal wash results. The authors concluded that regarding *M. bovis* diagnostics, both NS and guarded NP can be efficiently used in pneumonic calves. Our findings are consistent with theirs when considering NP: we obtained a proportion of positive agreement of 0.91 and a kappa coefficient of 0.84 when we compared NP with BAL in pneumonic calves. Van Driessche et al. (31) investigated the agreement of NP with BAL sampling in young veal and beef calves, and the kappa coefficient was 0.58 when direct culture of the samples was used. However, in NP they used a similar unguarded swab to that which we used in our NS. We obtained quite a similar kappa coefficient of 0.53 when NS culture results were compared with BAL results in pneumonic calves. Previously, Thomas et al. (32) compared NS with BAL in calves under 1 year of age and found that NS had a sensitivity of only 21%. Thus, NS was not predictive of *M. bovis* in the lower respiratory tract. Our results agree with those of van Driessche et al. (31) and Thomas et al. (32), suggesting that NS is not a sensitive sampling method to detect *M. bovis* in calves suffering from respiratory disease.

## Conclusions

Guarded NP at the group level is a sensitive and practical method to detect *M. bovis* in pneumonic calves. NS taken from young calves and analyzed by real-time PCR is a cost-efficient method to detect *M. bovis* in dairy herds, even if no *M. bovis* mastitis has been detected in the herd. We recommend that only calves under 6 months of age are sampled because in older calves, the prevalence of nasal shedding substantially decreases, although further study is needed to confirm this. Small herds in the control program are problematic because a reliable number of samples cannot be obtained. The suitability of new antibody ELISA tests in the Finnish control program should be evaluated. In the future, the effect of NS pooling on the sensitivity of PCR needs to be studied, as this would cost-efficiently allow a larger number of NS to be taken per herd. Finally, NP swabs appear to detect calves carrying *M. bovis* with a higher sensitivity than NS. Further studies are needed to verify this and determine the optimal use of these methods in the control program.

## Data Availability Statement

The original contributions presented in the study are included in the article/supplementary material, further inquiries can be directed to the corresponding author/s.

## Ethics Statement

Ethical review and approval was not required for the animal study because in this study normal sampling methods used routinely in bovine respiratory disease sampling was used and no ethical approval was thus required. Written informed consent was obtained from the owners for the participation of their animals in this study.

## Author Contributions

TP participated in the experimental design and method validation, analyzed the data, and wrote the article. NV was responsible for laboratory analyses and data management. VT conducted most of the sampling of recently infected dairy herds. SP was responsible for acquiring funding and project supervision and participated in experimental design. TA participated in the experimental design, method validation, data analysis, and project supervision. All authors contributed to revising the article and approved the submitted version.

## Funding

This study was funded by grant number 1876/312/2013 from the Ministry of Agriculture and Forestry of Finland.

## Conflict of Interest

The authors declare that the research was conducted in the absence of any commercial or financial relationships that could be construed as a potential conflict of interest.

## Publisher's Note

All claims expressed in this article are solely those of the authors and do not necessarily represent those of their affiliated organizations, or those of the publisher, the editors and the reviewers. Any product that may be evaluated in this article, or claim that may be made by its manufacturer, is not guaranteed or endorsed by the publisher.
